# Development of the oppression-based traumatic stress inventory: a novel and intersectional approach to measuring traumatic stress

**DOI:** 10.3389/fpsyg.2023.1232561

**Published:** 2023-10-24

**Authors:** Samantha C. Holmes, Daniel Zalewa, Chad T. Wetterneck, Angela M. Haeny, Monnica T. Williams

**Affiliations:** ^1^College of Staten Island, City University of New York, Staten Island, NY, United States; ^2^Behavioral Wellness Clinic, Tolland, CT, United States; ^3^Rogers Behavioral Health, Oconomowoc, WI, United States; ^4^Department of Psychiatry, Yale School of Medicine, New Haven, CT, United States; ^5^School of Psychology, University of Ottawa, Ottawa, ON, Canada

**Keywords:** trauma, racism, sexism, heterosexism, homophobia, minority stress, assessment

## Abstract

There is a growing body of literature demonstrating that experiences of oppression (e.g., racism, sexism, heterosexism, poverty) are associated with posttraumatic stress disorder symptoms. Traditional trauma assessments do not assess experiences of oppression and it is therefore imperative to develop instruments that do. To assess oppression-based traumatic stress broadly, and in an intersectional manner, we have developed the oppression-based traumatic stress inventory (OBTSI). The OBTSI includes two parts. Part A comprises open-ended questions asking participants to describe experiences of oppression as well as a set of questions to determine whether Criterion A for PTSD is met. Part B assesses specific posttraumatic stress symptoms anchored to the previously described experiences of oppression and also asks participants to identify the various types of discrimination they have experienced (e.g., based on racial group, sex/gender, sexual orientation, etc.). Clients from a mental health clinic and an undergraduate sample responded to the OBTSI and other self-report measures of depression, anxiety, and traditional posttraumatic stress (*N* = 90). Preliminary analyses demonstrate strong internal consistency reliability for the overall symptom inventory (α = 0.97) as well as for the four symptom clusters of posttraumatic stress symptoms in the DSM-5 (α ranging from 0.86 to 0.94). In addition to providing descriptive information, we also assess the convergent validity between the OBTSI and measures of anxiety, depression, and traditional posttraumatic stress and examine the factor structure. This study provides preliminary evidence that the OBTSI is a reliable and valid method of assessing oppression-based traumatic stress symptoms.

## Introduction

Oppression involves systemic discrimination that is specifically directed toward individuals with marginalized identities (Prilleltensky and Gonick, 1996). Occurring at the macro level of governmental policies and the micro level of internalized disdain for individuals solely due to their marginalized identity (Witherspoon et al., 2022), oppression results in cruel and unfair prevention of access to opportunities and freedoms that non-marginalized individuals can obtain with ease (Prilleltensky and Gonick, 1996). Oppression can occur across all marginalized identities including but not limited to race, ethnicity, sexual orientation, gender identity, socioeconomic status, immigration status, and ability. Marginalized individuals who experience oppression face significant barriers to accessing similar resources as their non-marginalized counterparts, such as mental or physical health care, employment, or even housing opportunities. In the context of mental or physical health, the barriers can revolve around a lack of trust in health and wellbeing professionals due to fear of continued victimization due to identity-based biases, biases which are commonly known to influence clinical decision making (Williams and Wyatt, 2015).

Psychological health outcomes are often poor for those who routinely experience oppression. In Phillips (1998), discussed the social and cultural contexts of domestic abuse faced by women, and the compounding negative effects caused by intersections of sexism, racism, and classism, while highlighting the importance of healthcare workers understanding this context in order to provide adequate care. Since then a growing body of literature has empirically demonstrated a link between experiences of oppression (e.g., racism, sexism, heterosexism, poverty) and posttraumatic stress symptoms. The majority of studies focus on the robust association between racism and posttraumatic stress symptoms (e.g., Loo et al., 2001; Cheng and Mallinckrodt, 2015; Watson et al., 2016; Sibrava et al., 2019) with additional studies examining the impact of sexism (e.g., Berg, 2006; Watson et al., 2016), heterosexism (e.g., Szymanski and Balsam, 2011; Bandermann and Szymanski, 2014; Dworkin et al., 2018), and poverty (e.g., Koenen et al., 2007; Golin et al., 2016; Holmes et al., 2021, 2022) on posttraumatic stress symptoms. Notably, the associations between experiences of oppression and posttraumatic stress symptoms persist longitudinally (Koenen et al., 2007; Flores et al., 2010; Cheng and Mallinckrodt, 2015; Golin et al., 2016; Sibrava et al., 2019; Holmes et al., 2022) and even when events that meet Criterion A for PTSD are statistically controlled for Loo et al. (2001), Szymanski and Balsam (2011), Bandermann and Szymanski (2014), Golin et al. (2016), Watson et al. (2016), and Holmes et al. (2022).

Recently, several measures have been developed to capture oppression-based traumatic stress. The Race-Based Traumatic Stress Scale (RBTSSS; Carter et al., 2013) and the Racial Trauma Scale (RTS; Williams et al., 2022a), self-report measures, and The UConn Racial/Ethnic Stress & Trauma Scale (UnRESTS; Williams et al., 2018a), a clinician-administered interview, were all developed to assess trauma symptoms associated with experiences of racism among people of color. These measures have been an important step in the field as they go beyond standard trauma assessments and allow for the accurate assessment of oppression-based traumatic stress. However, all three assess oppression-based traumatic stress that is specific to discrimination on the basis of race and ethnicity. The Trauma Symptoms of Discrimination Scale (TSDS; Williams et al., 2018b) assesses symptoms associated with a broad range of discriminatory experiences, while also highlighting how having multiple marginalized identities increases the risk for repeated experiences of trauma, thus increasing the risk of such symptoms (Williams et al., 2023). However, the items assess anxiety symptoms broadly, and do not specifically map onto the DSM-5’s criteria for PTSD. Thus, there is the need for an instrument that both assesses oppression-based traumatic stress in a broad and intersectional manner and also assesses symptoms specific to DSM-5’s PTSD. Doing so would allow for accurate assessment and referral to appropriate trauma-focused treatments for individuals who may fall through the diagnostic cracks of standard trauma assessments.

## Methods

### Development of the oppression-based traumatic stress inventory

Based on the UnRESTS, the OBTSI is a self-report questionnaire composed of two parts. In Part A, participants describe up to three examples of (a) major experiences of oppression they have experienced personally, (b) major experiences of oppression experienced by a loved one, and (c) microaggressions. They then respond to a series of questions designed to determine whether the oppression they experienced meets Criterion A for PTSD (i.e., DSM-5’s definition of trauma). In Part B, participants are presented with 25 items that describe DSM-5 PTSD symptoms and are asked to keep in mind their experiences of discrimination, as a whole, and indicate, in the past month, how much difficulty they have had with each symptom on a Likert scale ranging from 0 (not at all or almost never) to 4 (severely, or 7+ times a week). Finally, participants are asked to identify the type(s) of discrimination they have experienced (on the basis of racial group, sex/gender, sexual orientation, socioeconomic status, etc.) and, if they have selected multiple types, which type of discrimination they perceive to be a primary source.

The measure is scored by summing items 1–25 in Part B for a total OBTSI score, with potential scores ranging from 0 to 100. Items 1–5 represent Cluster B (re-experiencing), items 6–8 represent Cluster C (avoidance), items 9–19 represent Cluster D (negative alterations to mood and cognition), and items 20–25 represent Cluster E (hyperarousal). See Supplementary material for the full measure.

### Participants

The study sample comprised two groups, undergraduate students and clients at an outpatient mental health clinic. After IRB exemption was granted (Protocol #: 2023-0174-CSI), Sample 1 (*n* = 26) was recruited through a university’s undergraduate psychology department where students could earn course credit through participating in research studies via the program SONA. Sample 2 (*n* = 64) was recruited through an outpatient mental health clinic. Outpatients included those who were currently receiving treatment and all incoming clients who newly established treatment during the data collection period. Eligibility criteria included being over 18 years of age and able to read and write English. The combined sample comprised 90 participants. See Table 1 for demographic information.

**Table 1:** Demographics.

Descriptive		Sample 1	Sample 2
*N*		26	64
Age		20.84 (4.28)	38.08 (10.92)
Gender	Man	6	18
	Woman	19	45
	Transgender/Agender/Non-Binary	1	1
Ethnicity	Latine/Hispanic	5	6
	Non-Latine/Hispanic	21	58
Race	White/ European-American	5	37
	Black/African-American	10	12
	Asian/Asian-American	2	6
	Multiracial	0	2
	Other	9	6
	Did not disclose	0	1
Sexual identity	Heterosexual	23	45
	Sexual Minority	3	16
	Did not disclose	0	3

Given the goal of assessing the psychometric properties of Part B, participants who did not endorse experiences of oppression were excluded. Outpatient participants who were already receiving treatment had their data excluded if their BDI-II and BAI scores were obtained more than 3 months prior to completing the OBTSI, and newer measures were unobtainable.

### Measures

A comprehensive demographic form was completed to gather data including race, ethnicity, age, gender, religious affiliation, income, country of birth, sexual orientation, education level, and current occupation. The remaining measures are described below. All measures were completed by participants in Sample 2 via an online HIPAA compliant electronic medical records system as part of a clinical intake battery.

*The Posttraumatic Stress Disorder Checklist for DSM-5 (PCL-5;* Blevins et al., 2015) is a 20-item self-report measure that assesses PTSD symptoms according to the DSM-5. Participants are asked to rate the extent to which they were bothered by various problems within the past month relating to an index traumatic event identified by the Life Events Checklist for DSM-5 (Weathers et al., 2013) on a 5-point scale ranging from 0 (not at all) to 4 (extremely). The PCL-5 is a psychometrically sound measure of PTSD symptoms, demonstrating strong internal consistency, test–retest reliability, and convergent and discriminant validity (Blevins et al., 2015). The scale can be used to provide a provisional PTSD diagnosis. Although the PCL-5 was based on four DSM-5 criteria, recent studies have shown a 6- or 7-factor structure may be more appropriate (cf. Grau et al., 2019).

*The Beck Anxiety Inventory (BAI;* Beck et al., 1988) is a 21-item, 4-point scale that measures common symptoms of anxiety that participants have been experiencing within the last month, asking participants to indicate how much they have been bothered by that symptom from 0 (not at all) to 3 (severely). The measure is scored by summing items 1–21, with a maximum score of 63 indicating severe anxiety.

*The Beck Depression Inventory II (BDI-II;* Beck et al., 1987) is a 20 item, 4-point scale that consists of group statements related to common symptoms of depression such as sadness, guilt, loss of pleasure, worthlessness, etc. from 0 (not experiencing) to 3 (prominent display of symptom). The measure is scored by summing all of the items, with a maximum score of 63 indicating severe depression.

### Data analysis procedure

Cronbach’s alpha was computed for the total OBTSI scale and the four subscales representing each of the PTSD symptom clusters as per the DSM-5. Person’s correlations were conducted (two-tailed) for the correlations of psychopathology measures (PCL for Sample 1 and BAI and BDI-II for Sample 2) to the total OBTSI score and each of the 4 subscales representing the DSM-5 PTSD symptom clusters. For the correlation analyses, item-level missing data on the OBTSI and the PCL was addressed using available item analysis (Parent, 2013) providing that at least 80% of the items on the measure were completed (Downey and King, 1998). These analyses were conducted in SPSS version 27.

To better understand the structure of the OBTSI measure, an exploratory factor analysis (EFA) was conducted in Mplus version 8.4 using promax rotation (oblique) and full information maximum likelihood (FIML) to handle missing data. The number of factors was determined by a combination of theory and an examination of the eigenvalues, scree plot, and factor loadings.

## Results

### Descriptive statistics

The mean total OBTSI score for Sample 1 was 21.97 (19.58) and Sample 2 was 29.97 (25.74). The majority of participants (73.3%) reported experiencing discrimination on the basis of multiple identities (*M* = 2.77, SD = 1.81).

### Reliability

The OBTSI showed excellent internal consistency for the overall symptom inventory (α = 0.97). When examining the contribution of each item to the overall reliability, only the removal of item #21 improved the value of alpha, and only slightly (from 0.973 to 0.974). All items were highly correlated with the total score, with corrected item-total correlations ranging from 0.66–0.88, except for item #21, which was correlated at 0.42. The four symptom clusters of the symptom inventory also showed good internal consistency, with α ranging from 0.86 to 0.94 (see Table 2).

**Table 2:** Internal consistency reliability for the OBTSI and correlations among the full OBTSI and the symptom clusters.

	Cronbach’s Alpha	Cluster B	Cluster C	Cluster D	Cluster E
Cluster B (Re-experiencing)	0.94	–			
Cluster C (Avoidance)	0.86	0.88**	–		
Cluster D (Negative Mood / Cognition)	0.94	0.81**	0.83**	–	
Cluster E (Hyperarousal)	0.89	0.73**	0.70**	0.87**	–
OBTSI Total (All symptoms)	0.97	0.90**	0.90**	0.98**	0.90**

Sample sizes for Cronbach’s alphas ranged from 75 to 90; sample sizes for correlations ranged from 89 to 90. **Correlation significant at the 0.01 level (two-tailed).

### Correlational analyses

The total OBTSI was robustly correlated to each of the DSM-5 symptom clusters and each cluster was correlated to the other clusters, as shown in Table 2 in the combined sample.

Pearson’s correlations were conducted between the OBTSI and measures of psychopathology to establish convergent validity. In the student sample (Sample 1), the OBTSI total score was significantly correlated to the PCL-5 (*r* = 0.53, *p* = 0.006).

For the outpatient sample (Sample 2), measures of depression and anxiety (BAI and BDI-II) were correlated to the total OBTSI score and most DSM-5 PTSD symptom clusters (Clusters B-E). Table 3 depicts the correlations in Sample 2. Clusters B, C, D, and E, as well as the total OBTSI, score are positively correlated with the BAI, with correlation coefficients ranging from 0.43 to 0.51. Similarly, all symptom clusters and the total OBTSI score are positively correlated with the BDI-II, with correlation coefficients ranging from 0.39 to 0.61.

**Table 3:** Correlations between OBTSI and DSM-5 PTSD symptom cluster and measures of psychopathology in outpatients.

	BAI	BDI-II
Cluster B (Re-experiencing)	0.43**	0.39**
Cluster C (Avoidance)	0.47**	0.51**
Cluster D (Negative Mood/Cognition)	0.46**	0.59**
Cluster E (Hyperarousal)	0.49**	0.61**
OBTSI Total (All symptoms)	0.51**	0.58**

*N* = 56–60. **Correlation significant at the 0.01 level (two-tailed).

### Factor analytic findings

Examination of the eigenvalues, scree plot, and factor loadings suggested a 4-factor solution could be used (Table 4). Using a cut-point of 0.35 for each factor, all items loaded on one of the four factors, with no major cross-loading. Further, all factors were correlated (range = 0.54–0.64). The resultant factors did not completely map onto the four DSM-5 symptom clusters. Factor 1 included all of Cluster B (intrusion) items, all of Cluster C (avoidance) items and a single item from Cluster D – overall representing painful thoughts and memories of trauma. Factor 2 can be conceptualized as encompassing depressive symptoms as it included all of the Cluster D items that relate to anhedonia (i.e., feeling detached, loss of interest in previously enjoyable activities, difficulty experiencing positive emotions, emotional numbing), and nearly all of Cluster E (e.g., difficulty concentrating, sleep disturbances). Factor 3 included two items from Cluster D and one item from Cluster E and had the unifying theme of negative feelings about others and viewing the world as dangerous. Factor 4 included four items from Cluster D, with the major theme of negative thoughts about oneself, including self-blame and shame.

**Table 4:** Exploratory factor analysis.

Item	1	2	3	4
1. Reoccurring, unwanted distressing memories about discrimination- related experiences?	**0.751**	0.162	0.085	−0.034
2. Bad dreams or nightmares related to discrimination, or about feeling powerless or excluded?	**0.698**	0.031	0.133	−0.040
3. Feeling as if past discrimination-related event was happening to you all over again (like a flashback)?	**0.723**	0.198	−0.042	0.042
4. Getting very emotionally upset when reminded of discrimination-related experiences?	**0.806**	0.128	−0.034	0.108
5. Having physical reactions when reminded of discrimination- related experiences (e.g., stomachache, heart racing, shaking, sweating)?	**0.866**	0.007	0.142	0.072
6. Trying hard not to think about upsetting discriminatory experiences you have had?	**0.808**	0.067	−0.286	0.341
7. Avoiding activities, places, things, or situations that remind you of the discrimination-related experiences you have had?	**0.357**	0.075	0.221	0.296
8. Avoiding certain types of people because you worry they will behave in a discriminatory way (i.e., White people, law enforcement, bosses, etc.)?	**0.386**	−0.054	0.284	0.291
9. Difficulty remembering important parts of your experiences with discrimination?	−0.043	0.214	−0.070	**0.687**
10. Viewing yourself in a more negative way because of discrimination (e.g., “I should be a stronger person”)?	0.198	−0.041	0.093	**0.794**
11. Viewing others in a more negative way due to discrimination (e.g.,” I cannot trust White people,” “Religious people will not accept my sexual orientation,” “All men are dangerous”)?	0.050	−0.047	**0.954**	0.024
12. Viewing the world as a dangerous place because of your experiences with discrimination?	0.162	0.078	**0.591**	0.124
13. Blaming yourself for your experiences of discrimination, or for things that may have happened afterwards due to discrimination?	0.229	−0.024	0.123	**0.676**
14. Blaming others who were not involved for your experience, or for things that may have happened afterwards?	**0.384**	−0.046	0.261	0.123
15. Having ongoing negative feelings such as fear, horror, anger, guilt or shame because of your discrimination-related experiences?	0.256	0.091	0.323	**0.386**
16. Losing interest in activities you used to enjoy?	−0.011	**0.879**	0.065	−0.067
17. Feeling detached or cut-off from other people?	0.210	**0.705**	0.022	0.096
18. Difficulty experiencing positive feelings (like love or happiness)?	0.051	**0.829**	−0.136	0.110
19. Feeling emotionally numb?	0.011	**0.804**	−0.159	0.197
20. Acting irritable or (physically or verbally) aggressive?	−0.090	**0.790**	−0.011	0.097
21. Taking more risks or doing things that might harm you or others (e.g., driving recklessly, taking drugs, having unprotected sex)?	−0.093	**0.426**	0.157	0.028
22. Being overly alert or on-guard (e.g., checking to see who is around you, sitting in places where you can see everyone, etc.)?	−0.012	0.307	**0.421**	0.242
23. Being jumpy or more easily startled?	−0.055	**0.650**	0.218	0.073
24. Difficulty staying focused or concentrating?	0.226	**0.749**	−0.027	−0.027
25. Difficulty falling asleep or staying asleep?	0.244	**0.855**	0.085	−0.297

Factor loadings above 0.35 are in boldface. Item numbers for the DSM-5 clusters are Cluster B: 1–5, Cluster C: 6–8, Cluster D: 9–19, Cluster E: 20–25.

## Discussion

The aim of this study was to provide a preliminary validation of the OBTSI, and assess its utility as a clinical tool for measuring posttraumatic stress symptoms rooted in experiences of oppression. A strength of this study was the use of a relevant clinical sample in addition to the non-clinical sample for analysis.

### Reliability and validity

The OBSTI showed good reliability across the total scale and each individual subscale – Cluster B (re-experiencing), Cluster C (avoidance), Cluster D (negative alterations to mood/cognition), and Cluster E (hyperarousal). OBTSI scores demonstrated strong positive correlations with the PCL, BDI-II and BAI, furthering the literature of the link between posttraumatic stress symptoms, anxiety, and depression, and supporting the OBTSI’s validity as a measure for oppression-based traumatic symptoms. The correlation to the BDI-II was notably high, supporting existing literature that connects experiences of racism and feelings of helplessness (Madubata et al., 2018). Additionally, the moderately strong correlation between the PCL and OBTSI is noteworthy as it suggests that, despite the fact that both measures are assessing posttraumatic stress symptoms, by anchoring the symptoms to experiences of oppression, the OBTSI is assessing a different phenomenon and is not redundant with the PCL.

The reliability of item #21 was somewhat problematic, as it had a relatively low correlation to the adjusted total scare score. We recommend that the description of risky behaviors within item #21 be excluded from future versions of the OBTSI due to its stigmatizing nature, which may be doubly so in cases of marginalized participants, leading to low endorsement of this item. It is worthy of note that similar issues were noted in Grau et al. (2019) study on the PCL-5, and more recently in Williams and Zare’s (2022) study on the UnRESTS. Future research might include cognitive interviews to better understand how participants interpret the item and to elicit feedback on how the item might be reworded and subsequent empirical testing of whether editing the item in this manner improves the psychometric properties of the item in relation to the rest of the scale.

As noted, the four factors identified by the exploratory factor analysis did not match the DSM-5 clusters. This is unsurprising as factor analytic studies of the PTSD symptom clusters have been highly variable, with differing numbers of clusters identified and items disbursed differently than they are grouped in the DSM-5 (cf. Grau et al., 2019). In addition, the purpose of this measure was to identify reactions to oppression-based experiences, rather than to map on to the existing DSM-5 symptoms, which are based on clinical consensus and not statistical analysis or with oppression-based experiences as part of the consideration. Other studies focused on racial oppression have also found factor structures that differ from the suggested DSM-5 symptom dimensions (Carter et al., 2013; Roberson and Carter, 2022; Williams and Zare, 2022). These studies noted that those experiencing racial oppression demonstrate higher levels of dissociative symptoms compared to those without racial oppression (Williams et al., 2022a) and race-based trauma reactions were driven by depression, intrusive thoughts, anger, and low-self-esteem (Carter et al., 2013; Roberson and Carter, 2022).

The factors identified in the current study map onto factors identified on other measures of racial trauma. For example, Factor 1 in the current study includes both intrusive and avoidance symptoms which mirrors the factor labeled Intrusion on the RBTSSS (Carter et al., 2013). Similar to the RBTSSS’s Depression factor (Carter et al., 2013), the current study’s Factor 2 represents symptoms of depression (i.e., anhedonia, difficulty concentrating, sleep disturbances). Notably, depressive symptoms are a prominent feature of PTSD and also map on to previous factor analytic studies of PTSD symptoms outside the context of oppression-based traumatic stress (Grau et al., 2019). Factor 3, having negative feelings about others and viewing the world as dangerous, is similar to the RBTSSS’s Hypervigilance factor and the UnREST’s Negative Feelings About Others factor. Finally, the current study’s Factor 4 resembles the Low Self-Esteem factor of the RBTSSS (Carter et al., 2013) and the Self Loathing factor of the UnRESTS (Williams and Zare, 2022). It should be noted, however, that the current study included experiences that were not only linked to racial oppression, but included other identity-based oppression experiences and therefore may not perfectly replicate other studies that only assess race-based experiences.

### Implications

Experiences of oppression and accompanying oppression-based traumatic stress are relatively common. This paper describes a new intersectional measure of trauma focused on oppression-based traumatic stress that includes all PTSD symptoms in the DSM-5. Experiences of oppression most often involve discrimination on the basis of multiple marginalized identities, which highlights the importance of intersectional approaches to assessing oppression-based traumatic stress.

While there are other measures that assess oppression-based traumatic stress in a broad and intersectional manner (i.e., TSDS) or with items that map onto DSM-5 PTSD criteria (i.e., RTS, UnRESTS), the OBTSI is the first measure to do both. The OBTSI could be used as a screening tool to assess incoming clients for experiences of oppression as a way to begin a discussion about difficulties they may have faced throughout their lifetime, which may have led them to ultimately seek counseling or are worsening other conditions. It also could begin the process of psychoeducation and validation that a wide range of oppression-based experiences are important to mental health, extending the definition of what is traumatic early in the therapeutic process, based on the experience of the client. Additionally, inclusive measures allow clinicians to better engage in allyship behavior with clients whose background differs from theirs by demonstrating competency and understanding, which are known to increase rapport in and outside of clinical settings (Williams et al., 2022b). Finally, given that standard trauma assessments do not assess for experiences of oppression and associated posttraumatic stress, using the OBTSI may help clinicians identify clients who may benefit from trauma-focused treatment but who may have screened negative in standard trauma assessments.

### Limitations and future directions

Although many studies create clinical measures based on small, non-clinical samples, future research could address some of the concerns with this study’s sample size and make-up. While the current study demonstrated that OBTSI has strong psychometric properties, a larger sample size, with more robust numbers of specific marginalized identities across two time-points would improve the ability to perform more statistical analyses (e.g., a confirmatory factor analysis, convergent and divergent validity by group, test–retest reliability) and explore whether the identified factors generalize across areas of diversity. In addition, larger, more diverse samples could aid in determining the effects of oppression-based experiences on different types of intersectionality. While the study did include some clinical participants, studies looking at treatment-seeking and non-treatment seeking populations would be helpful to better understand the phenomenology of oppression-based traumatic stress, explore possible cutoffs for clinical needs, and further discussions in clinical settings about addressing these symptoms. To accomplish this goal, we are continuing data collection in both our university and outpatient settings. Additionally, we have initiated data collection in a domestic violence shelter and a series of partial hospitalization trauma treatment programs.

The current study is focused on assessing the psychometric properties of the OBTSI’s symptom inventory (i.e., Part B). Future research should be conducted focusing on Part A. For example, the OBTSI assesses oppression in an open-ended format. The data could be used to determine which types of oppressive experiences are most reliably linked to posttraumatic stress symptoms which could then be the basis of an inventory of oppressive events (similar to how the Life Events Checklist is used to assess traditional trauma exposure). While the symptoms of PTSD have stayed relatively the same over the past few decades, the types of events that constitute as traumatic and how different marginalized identities experience these events is largely unexplored. Part A of the OBTSI also includes a set of questions designed to determine whether Criterion A for PTSD is met. Future research could use data from these items to determine the frequency with which oppressive events meet Criterion A and the predictive validity of Criterion A for oppression-based traumatic stress (i.e., is meeting Criterion A associated with greater symptom severity and/or likelihood of meeting the other diagnostic criteria?).In addition to the above suggestions, future studies could focus on singular identities (based on gender, race, ethnicity, sexual orientation, religion, etc.), comparisons and similarities within these identity constructs (e.g., Black, Asian, and Native American; cisgender women and non-binary) and how intersectionality may play a role (i.e., when one possesses more than one marginalized identity).

## Conclusion

The OBTSI is a promising new measure that fills a needed gap in the research literature. It provides the mental health field with another tool that helps identify traumatic events based on an array of marginalized identities and evaluates the impact of symptoms associated with these experiences. This initial study determined that the OBTSI has demonstrated good reliability and validity for identifying oppression-based traumatic stress symptoms and we look forward to future research that will expand its application to other diverse marginalized identities and how intersectionality may influence a person’s experience.

## Data availability statement

The original contributions presented in the study are included in the article/Supplementary material, further inquiries can be directed to the corresponding author.

## Ethics statement

The studies involving humans were approved by College of Staten Island, City University of New York, Protocol #: 2023-0174-CSI. The studies were conducted in accordance with the local legislation and institutional requirements. The participants provided informed consent to participate in this study.

## Author contributions

The paper was jointly conceived by SH, MW, and CW. The instrument was developed by SH and MW. Data was collected by DZ, MW, and SH. Data was entered and cleaned by DZ and SH. Preliminary analyses were conducted by MW. The first draft of the manuscript was initially drafted by MW and DZ with edits made by SH and CW. Final analyses were conducted by AH and SH. All authors contributed to and approved the final manuscript.
