# Supplementary material for: Development of the oppression-based traumatic stress inventory: a novel and intersectional approach to measuring traumatic stress
Source: Front Psychol. 2023 Oct 24;14:1232561. doi: 10.3389/fpsyg.2023.1232561 (PMC10629001; doi:10.3389/fpsyg.2023.1232561)
Supplement: Supplementary file 1 [file Data_Sheet_1.PDF]

Oppression-Based Traumatic Stress Inventory (OBTSI): Part A – Experiences of Oppression

Discrimination is the unfair treatment of people based on parts of their identities, such as race, ethnicity, sexual orientation, gender, immigration status, or ability.

Definitions. Race: A group society puts a person in based on their appearance (e.g., skin color, features); Ethnicity: A person's culture based on their heritage (e.g., language culture); Gender: The cultural norms and expectations associated with biological sex; LGBTQ: A broad term encompassing individuals with marginalized sexual orientations and gender identities and expressions (e.g., gay, lesbian, bisexual, pansexual, transgender, gender non-binary).

Some people have difficult experiences over a lifetime that are manageable individually, but together they lead to feelings of stress or trauma. The following questions ask about some of your experiences of stress or trauma as it relates to discrimination. You may have been discriminated against or mistreated for reasons, such as your race, sexual orientation, gender, disability, faith, poverty, or a combination of reasons.

Experiences of Discrimination

1) Have you been impacted by discrimination, prejudice, oppression, and stigma? This could be something that someone either said or did to you that made you concerned about your safety or was very upsetting (for example: *hate crime, gender-based violence, harassment at work/school, victimization by law enforcement, assault, poor medical care, etc.*)
(*if you selected "no" to this item, please skip to #9)*

□ YES □ NO

2) Please briefly describe up to 3 events that were the most upsetting:

Event 1	Event 2	Event 3

Experiences of Discrimination Experienced by Loved Ones

3) Have you been impacted by discrimination as a result of something that happened to someone close to you? (for example: *workplace discrimination against a partner, wrongful imprisonment of a family member, deportation of relatives, poor medical care for a child, etc.*)
(*if you selected "no" to this item but selected "yes" to #1, please skip to #17; if you selected "no" to this item and also selected "no" to #1, skip to #24)*

□ YES □ NO

4) Please briefly describe up to 3 events that were the most upsetting:

Event 1	Event 2	Event 3

For the following questions, please keep in mind all of the experiences from above – both those you experienced personally, and those that were experienced by a loved one.	
**5) Was a loved one killed in any of the events described above?**	□ YES □ NO
**6) Did you feel like your life, or the life of a loved one, was threatened in any of the events described above?**	□ YES □ NO
**7) Were you, or a loved one, seriously *physically* injured as the result of any of the events described above?**	□ YES □ NO
**8) Were you, or a loved one, seriously *emotionally* injured as the result of any of the events described above?**	□ YES □ NO
**9) Were you, or a loved one, the victim of sexual violence in any of the events described above?**	□ YES □ NO
**10) Did any of the events described above include witnessing the death, threatened death, or serious injury of someone else?**	□ YES □ NO
**11) Did any of the events described above include repeated or extreme exposure to information, pictures, videos, or details relating to discriminatory violence (e.g., police brutality)?**<br><br>**If "yes", how were you exposed to this content?**<br>□ as part of your job (e.g., first responders collecting human remains, police offer repeatedly exposed to details of abuse)<br>□ as part of your day-to-day life (e.g., news, social media, word of mouth)<br>□ other: ___________________________________	□ YES □ NO

Experiences of Covert Discrimination & Microaggressions		
Often minorities and other groups treated poorly by society are the target of subtle discrimination, which we sometimes call microaggressions. Microaggressions include seemingly innocent or harmless comments, subtle or dismissive gestures, and tones that send degrading messages to a certain group (i.e., people of color, immigrants, women, LGBTQ people).		
**12) Have you ever experienced a microaggression?** □ YES □ NO<br>*(if you selected "no" to this item and also selected "no" to #1 and #9, you have completed this measure; if you have selected "no" to this item but selected "yes" to either #1 or #9, please skip to Part B)*		
**13) Please describe up to 3 recent examples:**		
Example 1	Example 2	Example 3

**Oppression-Based Traumatic Stress Inventory (OBTSI): Part B – Symptom Scale**
**When answering the following questions, keep in mind that discrimination is defined as being unfairly treated due to an individual characteristic of yourself that may be devalued by society (e.g., race/ethnicity, gender, sexual orientation, religion, etc.)**<br><br>**Experiencing discrimination can have a big impact, and sometimes people can feel stress due to that impact of discrimination in their daily lives. This can be caused by one very stressful experience of discrimination, or several smaller experiences of discrimination over the course of one's life. Based on these life experiences, answer the following questions. Please keep in mind that ratings should only reflect stress that was caused by discrimination.**<br><br>**Think about all of your experiences of discrimination as you answer the following questions.**<br><br>**In the <u>past month</u>, how much difficulty have you had with:**

		**Not at All**<br>(or almost never)	**A Little**<br>(or once a week or less)	**Somewhat**<br>(or 2-3 times a week)	**Very Much**<br>(or 4-6 times a week)	**Severely**<br>(or 7+ times a week)
**1)**	**Reoccurring, unwanted distressing memories about discrimination-related experiences?**	1	2	3	4	5
**2)**	**Bad dreams or nightmares related to discrimination, or about feeling powerless or excluded?**	1	2	3	4	5
**3)**	**Feeling as if a past discrimination-related event was happening to you all over again (like a flashback)?**	1	2	3	4	5
**4)**	**Getting very *emotionally* upset when reminded of discrimination-related experiences?**	1	2	3	4	5
**5)**	**Having *physical* reactions when reminded of discrimination-related experiences (e.g., stomachache, heart racing, shaking, sweating)?**	1	2	3	4	5
**6)**	**Trying hard not to think about upsetting discriminatory experiences you've had?**	1	2	3	4	5
**7)**	**Avoiding activities, places, things, or situations that remind you of the discrimination-related experiences you have had?**	1	2	3	4	5
**8)**	**Avoiding certain types of people because you worry they will behave in a discriminatory way (i.e., White people, law enforcement, bosses, etc.)?**	1	2	3	4	5
**9)**	**Difficulty remembering important parts of your experiences with discrimination?**	1	2	3	4	5

	Not at All<br><br>(or almost never)	A Little<br><br>(or once a week or less)	Somewhat<br><br>(or 2-3 times a week)	Very Much<br><br>(or 4-6 times a week)	Severely<br><br>(or 7+ times a week)
**10) Viewing yourself in a more negative way because of discrimination (e.g., "I should be a stronger person")?**	1	2	3	4	5
**11) Viewing others in a more negative way due to discrimination (e.g., "I can't trust White people" "Religious people won't accept my sexual orientation" "All men are dangerous")?**	1	2	3	4	5
**12) Viewing the world as a dangerous place because of your experiences with discrimination?**	1	2	3	4	5
**13) Blaming yourself for your experiences of discrimination, or for things that may have happened afterwards due to discrimination?**	1	2	3	4	5
**14) Blaming others who were not involved for your experience, or for things that may have happened afterwards?**	1	2	3	4	5
**15) Having ongoing negative feelings such as fear, horror, anger, guilt or shame because of your discrimination-related experiences?**	1	2	3	4	5
**16) Losing interest in activities you used to enjoy?**	1	2	3	4	5
**17) Feeling detached or cut-off from other people?**	1	2	3	4	5
**18) Difficulty experiencing positive feelings (like love or happiness)?**	1	2	3	4	5
**19) Feeling emotionally numb?**	1	2	3	4	5
**20) Acting irritable or (physically or verbally) aggressive?**	1	2	3	4	5
**21) Taking more risks or doing things that might harm you or others (e.g., driving recklessly, taking drugs, having unprotected sex)?**	1	2	3	4	5
**22) Being overly alert or on-guard (e.g., checking to see who is around you, sitting in places where you can see everyone, etc.)?**	1	2	3	4	5
**23) Being jumpy or more easily startled?**	1	2	3	4	5
**24) Difficulty staying focused or concentrating?**	1	2	3	4	5
**25) Difficulty falling asleep or staying asleep?**	1	2	3	4	5

	Not at All<br><br>(or almost never)	A Little<br><br>(or once a week or less)	Somewhat<br><br>(or 2-3 times a week)	Very Much<br><br>(or 4-6 times a week)	Severely<br><br>(or 7+ times a week)
**26) How much have these difficulties been bothering you? (all symptoms discussed so far)**	1	2	3	4	5

27) Please indicate the types of discrimination you have experienced in your lifetime (select all that apply):

□ Racial group (skin color, features, indigenous appearance)

□ Ethnic or cultural group **(if yes, what ethnic/cultural group has been the target for discrimination**: ________________)

□ Sex/Gender (especially being female or a woman)

□ Gender Identity (i.e., trans*, transgender, non-binary, etc.)

□ Sexual orientation (i.e., LGB status)

□ Ability Status (i.e., has a disability)

□ Weight or Size (i.e., overweight)

□ Religion (i.e., Islam, Judaism, Sikhs, etc.)

□ Poverty (especially lack of food, education, housing, basic needs)

□ Immigration Status (i.e., citizenship, undocumented status, etc.)

□ Age (i.e., for being either "too young" or "too old")

□ The above are just a few identities for which someone may experience discrimination. Please include any other areas not yet identified ____

(if you selected 2 or more categories) □ YES □ NO

28a) Do you feel that there was one type of discrimination you elected has been primary for you?

28b) If yes, please indicate which type of discrimination have been primary for you:

□ Racial group (skin color, features, indigenous appearance)

□ Ethnic or cultural group

□ Sex/Gender (especially being female or a woman)

□ Gender Identity (i.e., trans*, transgender, non-binary, etc.)

□ Sexual orientation (i.e., LGB status)

□ Ability Status (i.e., has a disability)

□ Weight or Size (i.e., overweight)

□ Religion (i.e., Islam, Judaism, Sikhs, etc.)

□ Poverty (especially lack of food, education, housing, basic needs)

□ Immigration Status (i.e., citizenship, undocumented status, etc.)

□ Age (i.e., for being either "too young" or "too old")

□ The above are just a few identities for which someone may experience discrimination. Please include any other areas not yet identified____
